# Monoclonal Antibody Therapy in Neuromyelitis Optica Spectrum Disorders: a Meta-analysis of Randomized Control Trials

**DOI:** 10.3389/fphar.2021.652759

**Published:** 2021-07-20

**Authors:** Fanxin Kong, Jianjun Wang, Haotao Zheng, Haobin Cai, Jun Hua, Liling Li

**Affiliations:** ^1^ Encephalopathy and Phycology Department, Shenzhen Traditional Chinese Medicine Hospital, ShenZhen, China; ^2^ Encephalopathy and Phycology Department, The Fourth Clinical Medical College of Guangzhou University of Chinese Medicine, ShenZhen, China; ^3^ Department of Clinical Pharmacy, Shenzhen Traditional Chinese Medicine Hospital, ShenZhen, China; ^4^ Department of Clinical Pharmacy, The Fourth Clinical Medical College of Guangzhou University of Chinese Medicine, ShenZhen, China

**Keywords:** neuromyelitis optica, inebilizumab, rituximab, eculizumab, satralizumab, tocilizumab, meta-analysis

## Abstract

**Background:** To update the efficacy and safety data of monoclonal antibodies for the treatment of neuromyelitis optica spectrum disorders (NMOSD) and explore the differences in the effect of treatment between patients seropositive and seronegative for AQP4-IgG. Methods: PubMed, Embase, and the Cochrane Library published up to July 2020 were searched for randomized controlled trials (RCTs) of monoclonal antibodies treatment (mAb) in patients with NMOSD. The primary outcome was the hazard ratio (HR) for relapse. The secondary outcomes included Expanded Disability Status Scale (EDSS) changes from baseline, adverse events (AEs), and serious adverse events (SAEs). A random-effects model was applied for the effect of heterogeneity among trials. Results: We included 603 patients (monoclonal antibody group, *n*=382, and control group, *n*=221) from seven RCTs. There were fewer relapses in the mAb group (HR=0.32, 95% CI: 0.23-0.46, *p*<0.001), as well as in the AQP4-IgG-seropositive patients (HR=0.18, 95% CI: 0.10–0.32, *p*<0.001), but not in AQP4-IgG-seronegative NMOSD. Similar results were observed when considering satralizumab only. The mAb had no impact on the changes in EDSS scores from baseline (WMD=−0.21, 95% CI: −0.50-0.09, *p*=0.176). The mAb did not lead to a higher frequency of AEs (OR=1.18, 95% CI: 0.70–1.98, *p*=0.529) or SAEs (OR=0.99, 95% CI: 0.63–1.56, *p*=0.975) compared with the control group. Conclusions: Compared to the control arm, monoclonal antibody therapy showed a significantly better outcome in restraining the HR for relapse among patients with NMOSD but insignificant effects in NMOSD patients with seronegative APQ4-IgG. The safety profile in each arm had no significant difference.

## Introduction

Neuromyelitis optica spectrum disorders (NMOSD) are devastating autoantibody-induced inflammatory diseases of the central nervous system that primarily affect the spinal cord, optic nerves, and brainstem, causing paralysis and blindness (Sellner et al., 2010; Kremer et al., 2014; Trebst et al., 2014; Wingerchuk et al., 2015). NMOSD encompasses a group of syndromes typically characterized by optic neuritis and/or acute myelitis, often in association with serum IgG autoantibodies directed against aquaporin-4 (AQP4-IgG), an astrocytic water channel (Papadopoulos and Verkman, 2012). The reported incidence and prevalence of NMOSD are dependent on geographical location and ethnicity; Asians and those of African ancestry are at increased risk, with high mortality rates reported in the latter (Wingerchuk, 2009; Sellner et al., 2010; Pittock and Lucchinetti, 2016; Mealy et al., 2018). Women are more affected than men, with a ratio of about 9:1 in adults and 3:1 in children (Wingerchuk et al., 2015). The median age of NMOSD onset is the late 30s (Sellner et al., 2010; Trebst et al., 2014).

The key point of the treatment of patients with NMOSD is to prevent relapse and reduce attack severity to lower the irreversible neurological impairments resulting from the successive attacks (Sellner et al., 2010; Wingerchuk et al., 2015). Several novel biological agents, including rituximab, eculizumab, inebilizumab, satralizumab, and tocilizumab, were applied in clinical trials to assess their effects on preventing NMOSD relapse. Azathioprine is effective for the management of relapses and disability in patients with NMOSD, but the adverse events are frequent and might limit its use (Espiritu and Pasco, 2019). Similar conclusions were reached for rituximab (Damato et al., 2016; Huang et al., 2019) and mycophenolate mofetil (Huang et al., 2019). Nevertheless, those conclusions are based on meta-analyses that included a wide variety of study types. To date, only one meta-analysis based on randomized controlled trials (RCTs) was published on the topic of NMOSD treatment (Xue et al., 2020), but it only included four RCTs, each investigating a different monoclonal antibody agent. Since the publication of that meta-analysis (Xue et al., 2020), three additional RCTs regarding the efficacy and safety of monoclonal antibody agents were completed and published (Tahara et al., 2020; Traboulsee et al., 2020; Zhang et al., 2020).

Therefore, the aim of this meta-analysis was to update the efficacy and safety data of monoclonal antibodies for the treatment of NMOSD and explore the differences in the effect of treatment between patients seropositive and seronegative for AQP4-IgG.

## Materials and Methods

### Literature Search

This meta-analysis was conducted according to the Preferred Reporting Items for Systematic Reviews and Meta-Analyses (PRISMA) guidelines (Selcuk, 2019). We started by searching relevant articles according to the PICOS principle (Aslam and Emmanuel, 2010), followed by screening on the basis of the inclusion criteria: 1) Population: NMOSD; 2) Interventions: using monoclonal antibody therapy; 3) Comparison: different treatment or placebo; 4) Study: RCTs; and 5) Language: English. PubMed, Embase, and the Cochrane Library were searched for available papers published up to July 2020, using the MeSH terms “Neuromyelitis Optica” as well as relevant key words such as “monoclonal antibody” and “randomized controlled trial.”

### Data Extraction

The study characteristics (authors, year of publication, site of the study population, blinding methodology, and age of the patients), treatment parameters (sample size, median follow-up time, treatment, and control agents), and outcomes (hazard ratio (HR) for relapse, Expanded Disability Status Scale (EDSS) changes from baseline, adverse events (AEs), and serious adverse events (SAEs)) were extracted by two authors, independently. The discrepancies were solved by discussion.

### Data Synthesis

For outcome assessments, the HRs of relapse and their 95% confidence intervals (CIs) were extracted from the Kaplan-Meier survival curves in each study. To account for more conservative results, studies that reported relative risks (RRs) were not considered in this pooled analysis. EDSS score changes from baseline were reported in four of the trials, and the number of patients in each arm, as well as the mean change with its standard deviation, were used for analysis. The numbers of cases of AEs and SAEs in each arm were used to conclude the safety results.

### Quality of the Evidence

The level of evidence of all RCTs was assessed independently by two authors according to the Cochrane Handbook and NOS criteria (Lo et al., 2014; Higgins et al., 2019). Discrepancies in the assessment were resolved through discussion until a consensus was reached.

### Statistical Analysis

All analyses were performed using the STATA SE 14.0 software (StataCorp, College Station, Texas, United States). The effects and corresponding 95% CIs were used to compare the outcomes. Weighted mean differences (WMDs) were used for the changes in EDSS. AEs and SAEs were analyzed in terms of odds ratios (ORs). Statistical heterogeneity among the studies was calculated using Cochran’s Q test and the I^2^ index (Higgins et al., 2019). To avoid the effect of heterogeneity between each study, regardless of the results of Cochran’s Q test and the I^2^ index, random effect models were applied for the pooled analyses. We did not assess potential publication bias by funnel plots and Egger’s test because the number of studies included in every meta-analysis was smaller than 10, in which case the funnel plots and Egger’s test could yield misleading results and are not recommended (Higgins et al., 2011; Higgins et al., 2019).

### Role of the Funding Source

The funder of the study had no role in study design, data collection, data analysis, data interpretation, or writing of the report. The corresponding author had full access to all the data in the study and had final responsibility for the decision to submit for publication.

## Results

### Study Selection Process

**FIGURE 1:**
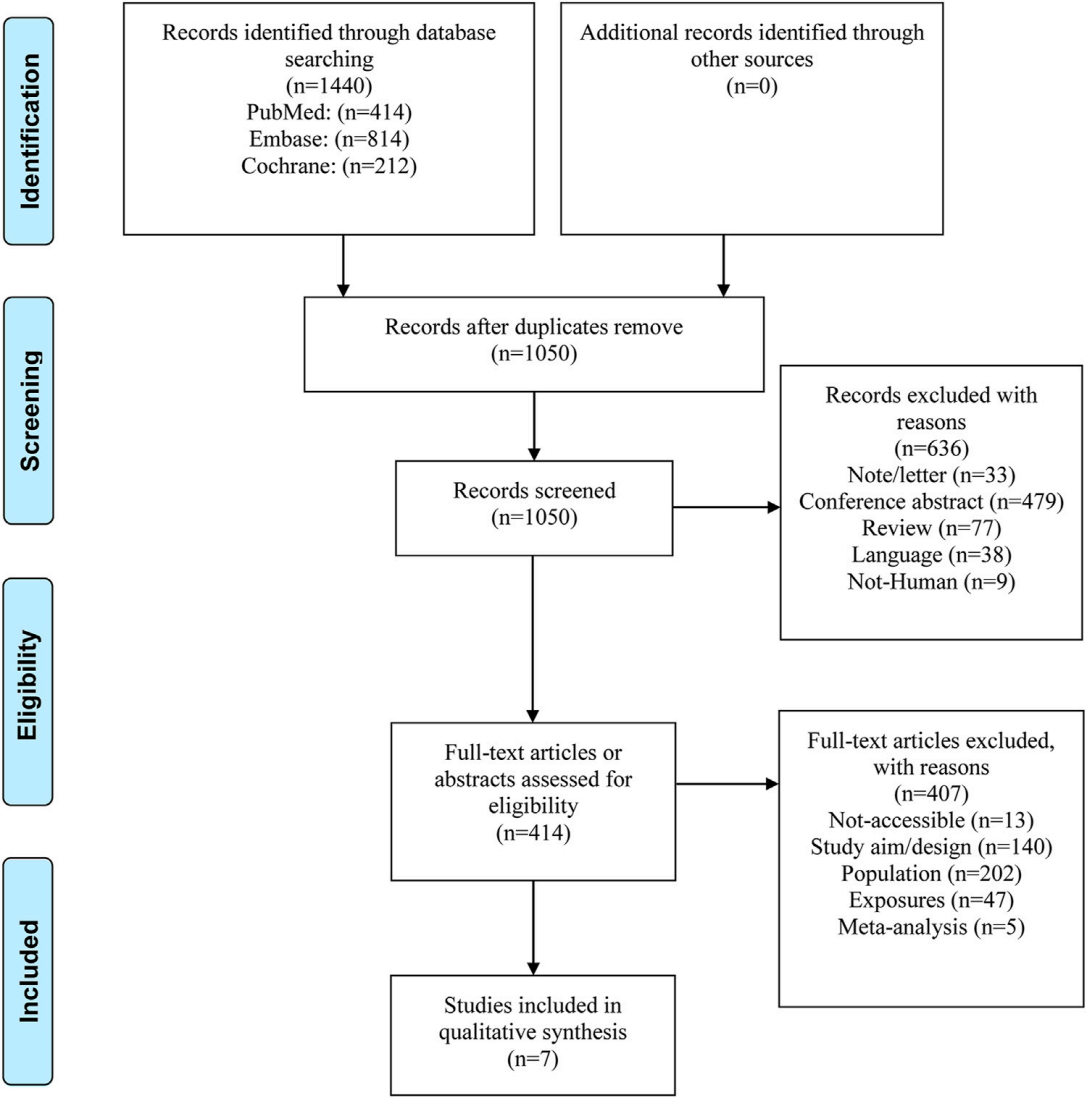
PRISMA 2009 Flow Diagram.

Figure 1 shows the study selection process. A total of 1,440 records were initially identified. After removing the duplicates, 1,050 were screened, and 636 were excluded. The 414 remaining full-text papers were assessed for eligibility and 407 were excluded (no accessible full-text, *n* = 13; study aim/design, *n* = 140; population, *n* = 202; exposures, *n* = 47; and meta-analyses, *n* = 5).

Finally, seven RCTs were selected, encompassing 382 patients with the study drug and 221 controls (Nikoo et al., 2017; Cree et al., 2019; Pittock et al., 2019; Yamamura et al., 2019; Tahara et al., 2020; Traboulsee et al., 2020; Zhang et al., 2020) (Table 1). There were five double-blind RCTs and two open-label ones. One study examined inebilizumab (Cree et al., 2019), two examined rituximab (Nikoo et al., 2017; Tahara et al., 2020), one examined eculizumab (Pittock et al., 2019), two examined satralizumab (Yamamura et al., 2019; Traboulsee et al., 2020), and one examined tocilizumab (Zhang et al., 2020). Five studies used a placebo as control (Cree et al., 2019; Pittock et al., 2019; Yamamura et al., 2019; Tahara et al., 2020; Traboulsee et al., 2020), while two used azathioprine (Nikoo et al., 2017; Zhang et al., 2020).

**TABLE 1:** Characteristics of the studies.

Author, Year	Localization	Blinding	Group (*N*)		Age, year		Any adverse events, *n*	
			Treatment	Control	Treatment	Control	Treatment	Control
Cree, 2019 Cree et al. (2019)	99 sites in 25 countries	Double-blind	Inebilizumab (174)	Placebo (56)	43.0 ± 11.6	42.6 ± 13.9	125	41
Nikoo, 2017 Nikoo et al. (2017)	1 hospital in Iran	Open-label	Rituximab (40)	Azathioprine (46)	34.33 ± 9.04	32.11 ± 9.36	NR	NR
Pittock, 2019 Pittock et al. (2019)	70 sites in 18 countries	Double-blind	Eculizumab (96)	Placebo (47)	43.9 ± 13.32	45.0 ± 13.29	88	43
Tahara, 2020 Tahara et al. (2020)	8 sites in Japan	Double-blind	Rituximab (19)	Placebo (19)	53.0 (42.0–58.0)	47.0 (37.0–65.0)	17	17
Traboulsee, 2020 Traboulsee et al. (2020)	44 sites in 13 countries	Double-blind	Satralizumab (63)	Placebo (32)	45.3 ± 12.0	40.5 ± 10.5	58	24
Yamamura, 2019 Yamamura et al. (2019)	34 sites in 11 countries	Double-blind	Satralizumab (41)	Placebo (42)	40.8 ± 16.1	43.4 ± 12.0	37	40
Zhang, 2020 Zhang et al. (2020)	6 hospital in China	Open-label	Tocilizumab (59)	Azathioprine (59)	48.1 ± 13.4	45.3 ± 14.5	57	56

### Relapse of NMOSD

**FIGURE 2:**
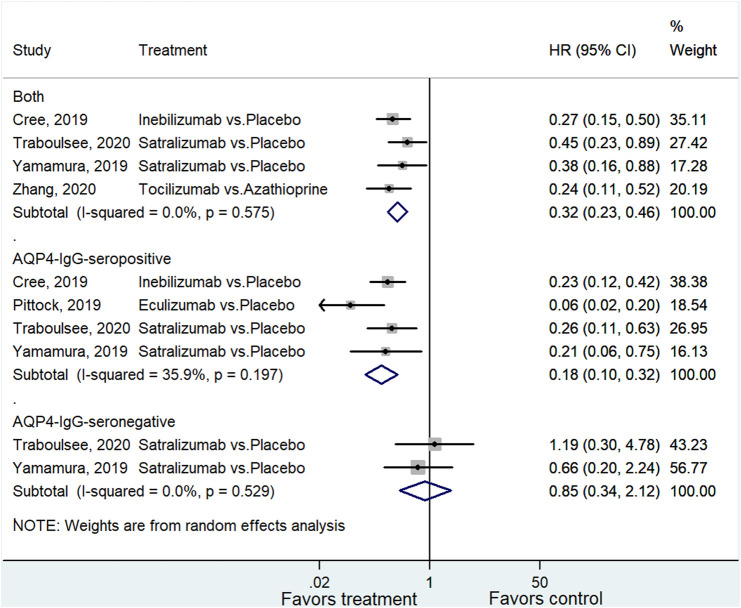
Forest plot of HR for relapse in all NMOSD, AQP4-IgG-seropositive NMOSD, and AQP4-IgG-seronegative NMOSD patients.

For studies that reported the HR of relapse for all NMOSD patients (Cree et al., 2019; Yamamura et al., 2019; Traboulsee et al., 2020; Zhang et al., 2020). The pooled analysis showed a significantly better outcome in the treatment group (HR = 0.32, 95% CI: 0.23–0.46, *p* < 0.001; I^2^ = 0.0%, P_heterogeneity_ = 0.575). Subgroup analysis for AQP4-IgG-seropositive and negative patients indicated a positive association between treatment and favorable outcome among patients with AQP4-IgG-seropositive NMOSD (HR = 0.18, 95% CI: 0.10–0.32, *p* < 0.001; I^2^ = 35.9%, P_heterogeneity_ = 0.197), whereas the association with AQP4-IgG-seronegative NMOSD was not significant (HR = 0.85, 95% CI: 0.34–2.12, *p* = 0.729; I^2^ = 0.0%, P_heterogeneity_ = 0.529) (Figure 2 and Supplementary Table 1).

### Subgroup Analysis of Satralizumab

**FIGURE 3:**
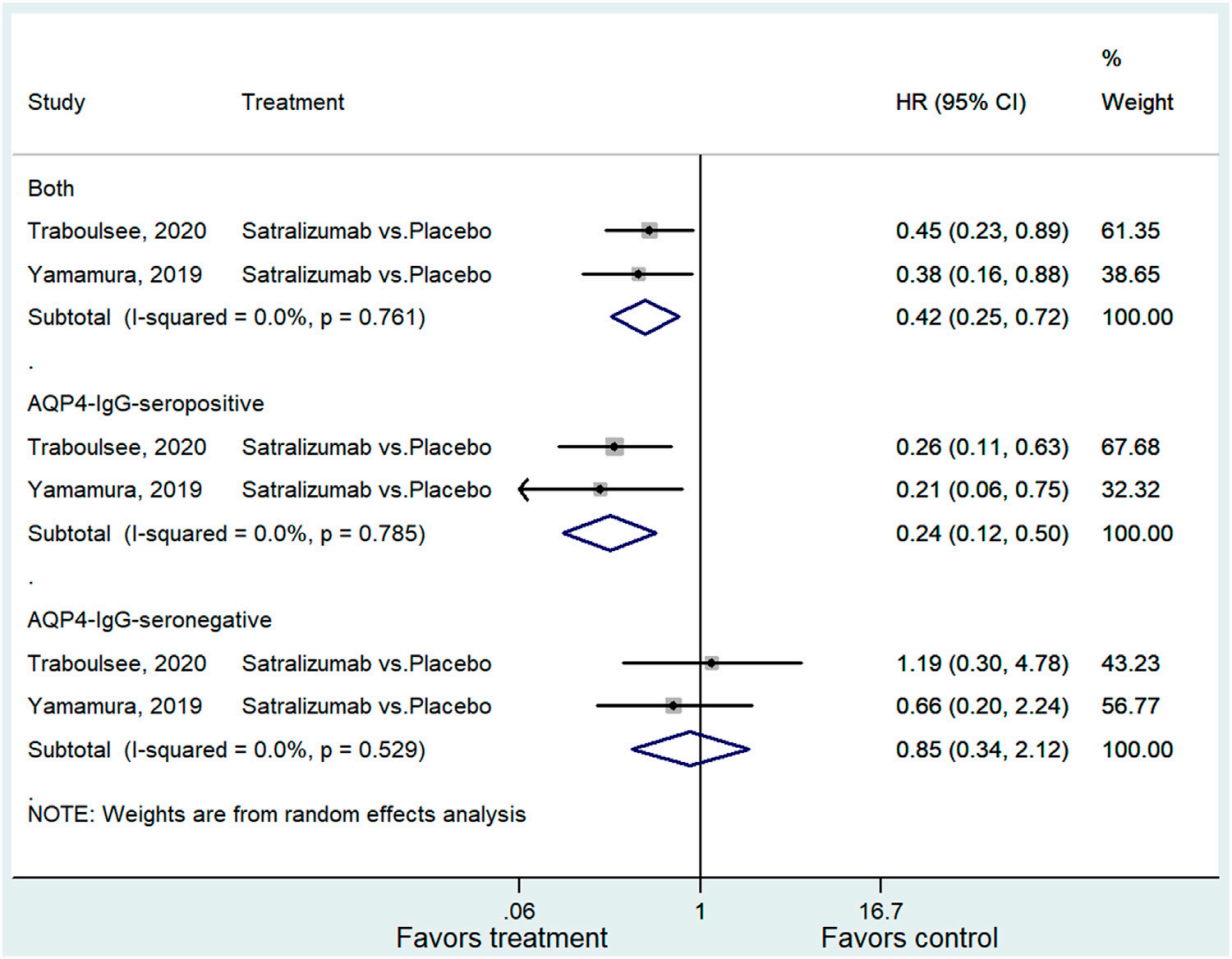
Subgroup analysis for the efficacy of satralizumab in each group of patients.

Two studies examined satralizumab on the relapse of NMOSD (Yamamura et al., 2019; Traboulsee et al., 2020). The pooled analysis showed a significantly better outcome in the satralizumab group (HR = 0.42, 95% CI: 0.25–0.72, *p* = 0.001; I^2^ = 0.0%, P_heterogeneity_ = 0.761). Subgroup analysis for AQP4-IgG-seropositive and negative patients indicated a positive association between treatment and favorable outcome among patients with AQP4-IgG-seropositive NMOSD (HR = 0.24, 95% CI: 0.12–0.50, *p* < 0.001; I^2^ = 0.0%, P_heterogeneity_ = 0.785), whereas the association with AQP4-IgG-seronegative NMOSD was not significant (HR = 0.85, 95% CI: 0.34–2.12, *p* = 0.719; I^2^ = 0.0%, P_heterogeneity_ = 0.529) (Figure 3 and Supplementary Table 1).

### Changes in EDSS Scores

**FIGURE 4:**
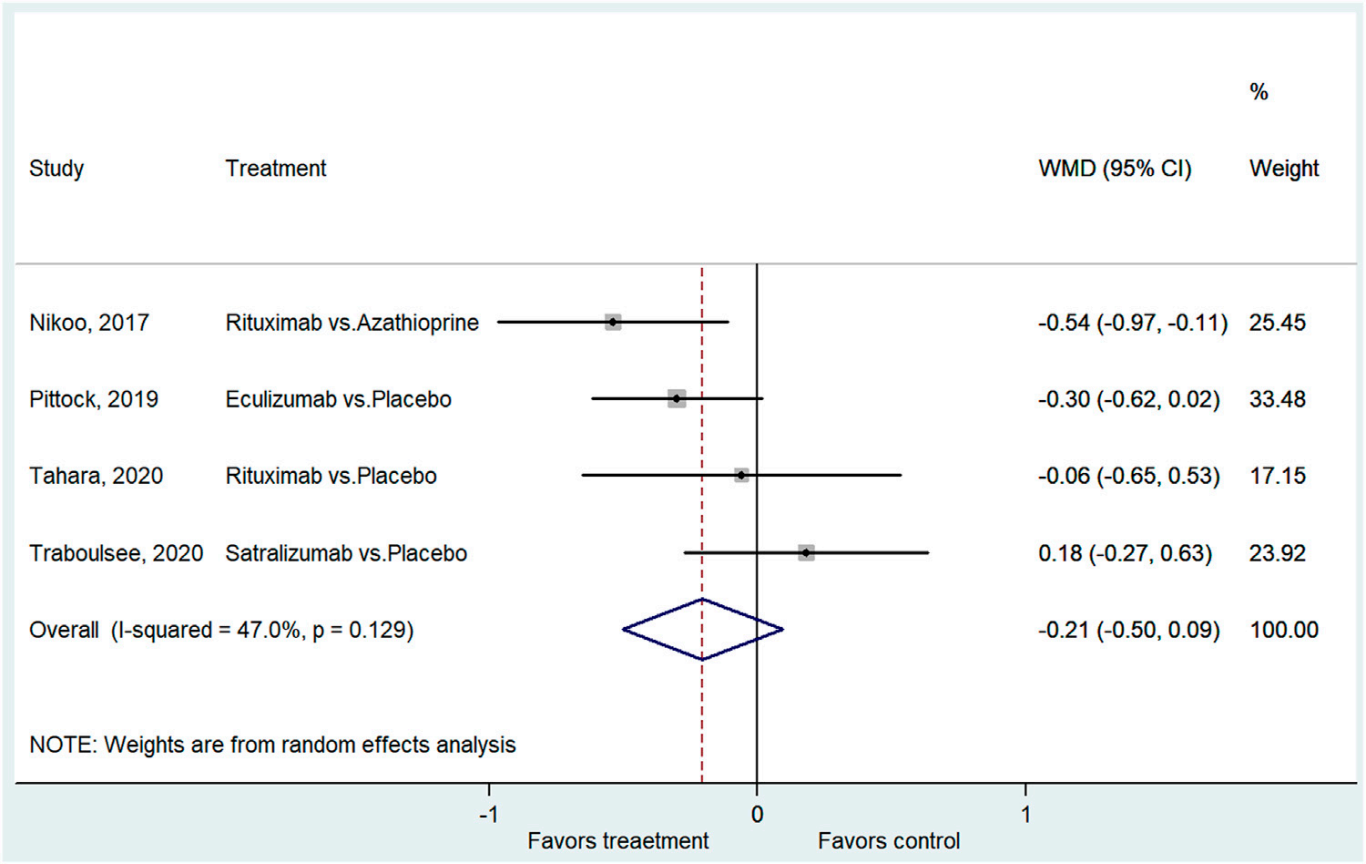
Forest plot of EDSS score change from baseline.

Four studies reported the changes in EDSS (Nikoo et al., 2017; Pittock et al., 2019; Tahara et al., 2020; Traboulsee et al., 2020). The treatment had no impact on the changes in EDSS scores from baseline (WMD = −0.21, 95% CI: −0.50–0.09, *p* = 0.176; I^2^ = 47.0%, P_heterogeneity_ = 0.129) (Figure 4 and Supplementary Table 1).

### AEs and SAEs

**FIGURE 5:**
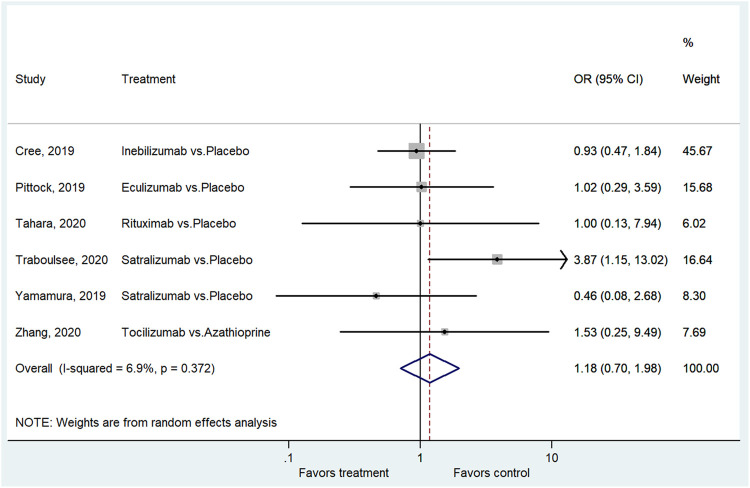
Forest plot of AEs.

**FIGURE 6:**
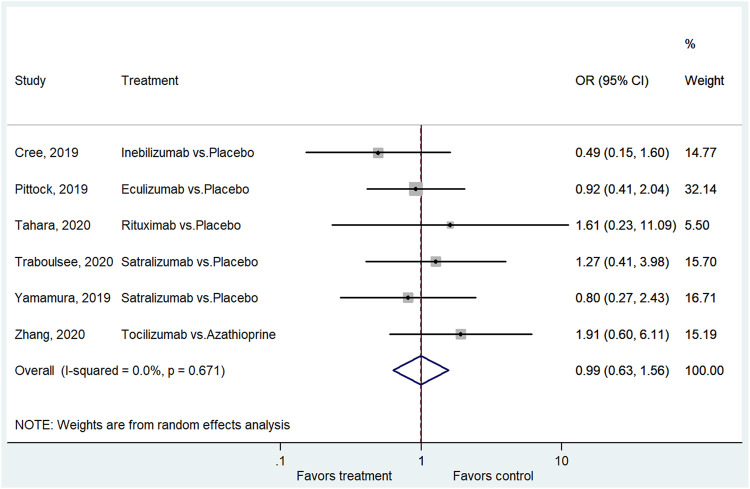
Forest plot of the SAEs.

Six studies reported the AEs (Cree et al., 2019; Pittock et al., 2019; Yamamura et al., 2019; Tahara et al., 2020; Traboulsee et al., 2020; Zhang et al., 2020). The treatment did not lead to a higher frequency of AEs compared with the control group (OR = 1.18, 95%CI: 0.70–1.98, *p* = 0.529; I^2^ = 6.9%, P_heterogeneity_ = 0.372) (Figure 5 and Supplementary Table 1). Six studies reported the SAEs (Cree et al., 2019; Pittock et al., 2019; Yamamura et al., 2019; Tahara et al., 2020; Traboulsee et al., 2020; Zhang et al., 2020). The treatment did not lead to a higher frequency of SAEs compared with the control group (OR = 0.99, 95% CI: 0.63–1.56, *p* = 0.975; I^2^ = 0.0%, P_heterogeneity_ = 0.671) (Figure 6 and Supplementary Table 1).

### Quality Assessment

**FIGURE 7:**
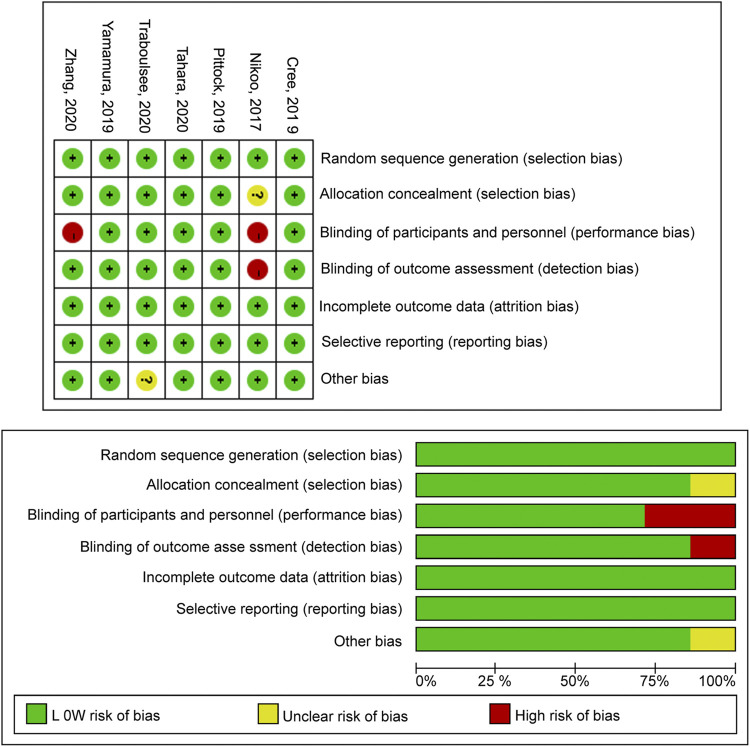
Cochrane criteria for quality of RCT.

Four studies had a low risk of bias for each of the items of the Cochrane tool (Cree et al., 2019; Pittock et al., 2019; Yamamura et al., 2019; Tahara et al., 2020). Nikoo et al. (2017) had an unclear risk of bias regarding allocation concealment and high risks of bias for blinding of participants and personnel and blinding of outcome assessment. Traboulsee et al. (2020) carried an unclear risk of bias for other biases. Zhang et al. (2020) had a high risk of bias for blinding of participants and personnel. The details were summarized in Figure 7.

### Sensitivity Analysis

**FIGURE 8:**
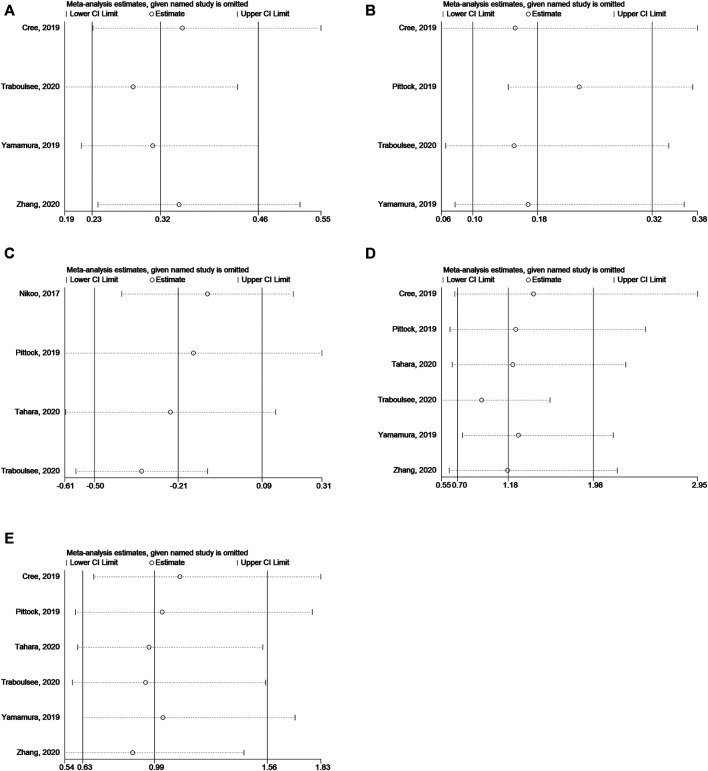
**(A)** Sensitivity analysis for HR of relapse in NMOSD patients. **(B)** Sensitivity analysis for HR of relapse in NMOSD patients with AQP4-IgG-seropositive. **(C)** Sensitivity analysis for EDSS score change in NMOSD patients. **(D)** Sensitivity analysis for odds of AEs. **(E)** Sensitivity analysis for odds of SAEs.

The sensitivity analysis showed that none of the studies significantly affected the results of the above analyses (Figures 8A–E).

## Discussion

Monoclonal antibodies can be used for the management of NMOSD, but evidence from meta-analyses is rare. Therefore, this meta-analysis aimed to update the efficacy and safety data of monoclonal antibodies for the treatment of NMOSD and explore the differences in the effect of treatment between patients seropositive and seronegative for AQP4-IgG. The results indicate that compared to the control arm, monoclonal antibody therapy showed a significantly better outcome in restraining the HR for relapse among patients with NMOSD. The effects in patients with seronegative APQ4-IgG NMOSD patients were not significant. The safety profile in each arm had no significant difference.

In NMOSD, the poor outcomes are due to the repeated relapses that lead to progressive neurologic impairment (Birnbaum and Kerr, 2008; Jacob et al., 2009; Jiao et al., 2013; Hinson et al., 2016). Two previous meta-analyses of rituximab for NMOSD showed that rituximab could decrease the relapse events of NMOSD and alleviate the neurological disability of the patients (Damato et al., 2016; Huang et al., 2019). A meta-analysis of four RCTs showed that the monoclonal antibodies decreased the annualized relapse rate, on-trial relapse risk, EDSS score, and the occurrence of SAEs compared with the control treatment (Xue et al., 2020). In addition, the benefits were observed for AQP-4-positive patients. When adding three additional RCTs, similar results are still observed. Those effects are mainly attributable to B cell depletion (De Romeuf et al., 2008).

Azathioprine has been shown to be effective for the management of relapses and disability in patients with NMOSD, but the adverse events are frequent and might limit its use (Espiritu and Pasco, 2019). Despite this efficacy, the monoclonal antibodies were still more effective than azathioprine and mycophenolate mofetil, as for the comparison between the monoclonal antibodies and the placebo. In addition, the monoclonal antibodies had a tolerability profile that was more advantageous than for azathioprine. Similar results were observed for mycophenolate mofetil (Huang et al., 2019). Therefore, monoclonal antibodies are probably a better option than other immunomodulatory drugs for the management of NMOSD. Nevertheless, their costs are high, and cost-benefit analyses should be performed. In addition, two meta-analyses of rituximab raised cautions regarding its use as a first-line agent in NMOSD because of the tolerability profile (Damato et al., 2016; Huang et al., 2019). Of note, those meta-analyses examined all kinds of study design together, leading to high heterogeneity. In the present meta-analysis, the AE and SAE profile were not significantly worse than that of the control arm, but they should be taken with caution because of the two studies that used azathioprine as a control group, probably increasing the numbers of safety events in the control arm. Depleting B cells carries a theoretical risk of increased cancers and infection (Radaelli et al., 2016; Hauser et al., 2017), which should be examined in future studies.

Of note, NMOSD encompasses a group of syndromes that share optic neuritis and/or acute myelitis as their manifestation, but the patients can be seropositive or seronegative for AQP4-IgG, supporting the heterogeneity of the conditions included in NMOSD (Papadopoulos and Verkman, 2012), and particularly in AQP4-IgG-negative NMOSD (Wingerchuk et al., 2015). Clinically, patients with AQP4-IgG-positive or -negative NMOSD cannot be distinguished (Wingerchuk et al., 2007; Sepulveda et al., 2016; Weinshenker and Wingerchuk, 2017). Previous studies (Cree et al., 2019; Pittock et al., 2019; Yamamura et al., 2019; Traboulsee et al., 2020), as the present meta-analysis, support the use of monoclonal antibodies for AQP4-IgG-positive NMOSD. Still, monoclonal antibody therapy was tried in AQP4-IgG-negative NMOSD (Yamamura et al., 2019; Traboulsee et al., 2020). Traboulsee et al. (2020) included both AQP-IgG-negative and positive patients in an attempt to encompass the whole spectrum of NMOSD, and they reported no benefit of satralizumab in such patients. Such results are supported by the SAkuraStar trial by Yamamura et al. (2019). Still, they attributed this lack of efficacy to the small sample size (the proportion of negative patients was capped to represent their frequency in the population (Wingerchuk et al., 2007; Sepulveda et al., 2016)) and to the heterogeneity of AQP4-IgG-negative NMOSD (Wingerchuk et al., 2015). In addition, their study was not powered to examine efficacy in AQP4-IgG-negative NMOSD patients (Traboulsee et al., 2020). Studies that will include more patients with AQP4-IgG-negative NMOSD or studies that will better characterize the pathogenesis of AQP4-IgG-negative NMOSD could provide more definitive answers about the management of such patients.

Importantly, all safety data included in the present meta-analysis and reported by the included studies are short-term safety data. The long-term adverse events associated with monoclonal antibody therapy vary according to the target of the antibody used but generally include infections, cancers, autoimmune diseases, and organ-specific toxicity (e.g., cardiotoxicity and lung toxicity) (Hansel et al., 2010; Lu et al., 2020). Among the antibodies included in the meta-analysis, rituximab is the one with the longest market life. The late-onset AEs of rituximab include neutropenia, immune compromise, infections, leukoencephalopathy, viral reactivation, intestinal perforation, and pneumonitis (Ram et al., 2009). For the other antibodies included here, fewer data are available. Tocilizumab might increase the risk of infection and cancer (Jones and Panova, 2018). Real-life studies are necessary to determine the long-term safety of these antibodies.

This study has limitations. Although we included all newly published RCTs in our meta-analysis, the number of RCTs is still limited. Nevertheless, the sensitivity analysis showed robust results of monoclonal antibody therapy in the treatment of patients with NMOSD. In addition to the small number of RCTs, the seven RCTs covered five different monoclonal antibodies, and the controls were either a placebo or a comparator drug. The drug mechanism of each monoclonal antibody therapy may vary significantly, which will inevitably increase the heterogeneity of the meta-analysis. Therefore, we applied the random-effect model in all pooled analyses to minimize this effect.

In conclusion, compared with the control arm, monoclonal antibody therapy showed a significantly better outcome in restraining the HR for relapse among patients with NMOSD, despite the fact that the effects in patients with NMOSD with seronegative APQ4-IgG were not significant. The safety profile in each arm had no significant difference, which indicates a satisfying safety outcome. More RCTs are needed for each monoclonal antibody individually. A network meta-analysis with abundant studies comparing different monoclonal antibodies is encouraged.

## Data Availability

The original contributions presented in the study are included in the article/supplementary material, further inquiries can be directed to the corresponding author.
