# Supplementary material for: Monoclonal Antibody Therapy in Neuromyelitis Optica Spectrum Disorders: a Meta-analysis of Randomized Control Trials
Source: Front Pharmacol. 2021 Jul 20;12:652759. doi: 10.3389/fphar.2021.652759 (PMC8329455; doi:10.3389/fphar.2021.652759)
Supplement: Supplementary file 1 [file Table1.docx]

**Supplementary Table 1.** Comparisons of the treatment vs. control arms

|  | N | ES (95%CI) | P | I-square, % | P (Heterogeneity) |
| --- | --- | --- | --- | --- | --- |
| HR for relapse |  |  |  |  |  |
| Both | 4 | 0.321 (0.226, 0.458) | <0.001 | 0 | 0.575 |
| AQP4-IgG-seropositive | 4 | 0.182 (0.102, 0.324) | <0.001 | 35.9 | 0.197 |
| AQP4-IgG-seronegative | 2 | 0.852 (0.343, 2.116) | 0.729 | 0 | 0.529 |
| Satralizumab |  |  |  |  |  |
| Both | 2 | 0.422 (0.248, 0.716) | 0.001 | 0 | 0.761 |
| AQP4-IgG-seropositive | 2 | 0.243 (0.118, 0.497) | <0.001 | 0 | 0.785 |
| AQP4-IgG-seronegative | 2 | 0.852 (0.343, 2.116) | 0.719 | 0 | 0.529 |
| EDSS change from baseline | 4 | -0.205 (-0.503, 0.092) | 0.176 | 47.0 | 0.129 |
| PP | 1 | -0.540 (-0.968, -0.112) | 0.013 | . | . |
| ITT | 3 | -0.101 (-0.406, 0.204) | 0.517 | 32.4 | 0.228 |
| AEs | 6 | 1.180 (0.705, 1.977) | 0.529 | 6.9 | 0.372 |
| SAEs | 6 | 0.993 (0.631, 1.561) | 0.975 | 0 | 0.671 |
